# When a neglected tropical zoonotic disease emerges in non-endemic countries: need to proactively fill the unveiled knowledge gaps towards human monkeypox among the Lebanese population

**DOI:** 10.1186/s40545-023-00544-1

**Published:** 2023-03-07

**Authors:** Dalal Youssef, Edmond Abboud, Malak Kawtharani, Zahraa Zheim, Nisrine Abou Arrage, Janet Youssef

**Affiliations:** 1 Institut de Santé Publique, d’épidémiologie et de Développement (ISPED) School of Public Health, UMR_S 1219, Research Center Bordeaux Population Health (BPH), Bordeaux University, Bordeaux, France; 2 Clinical Trial Program, Ministry of Public Health, Beirut, Lebanon; 3 Lebanese Higher Institute of Technical and Professional (IPNET), Bir Hassan, Ministry of Education, Beirut, Lebanon; 4 Ministry of Public Health, Beirut, Lebanon; 5 Antonine University, Riyak, Lebanon; 6 Al Zahraa Hospital Medical Center, Beirut, Lebanon

**Keywords:** Knowledge, Assessment, Monkeypox, Lebanese population, Non-endemic countries

## Abstract

**Introduction:**

The ongoing multi-country outbreak of monkeypox (MPX) that emerges in non-endemic areas is a rare and unprecedented event that has sparked a widespread public health concern. Lebanon has reported four confirmed cases of MPX so far. Since good knowledge about the MPX virus and its associated disease is paramount for helping the Lebanese population prepare for a possible outbreak, therefore, it is important to assess their current level of knowledge regarding MPX and to identify its associated factors to highlight any knowledge gaps that need to be filled.

**Methods:**

An online cross-sectional study was conducted over the first 2 weeks of August 2022 among adults aged 18 years and above recruited from all Lebanese provinces using a convenience sampling technique. An anonymous, Arabic, self-reported questionnaire covering all main aspects of knowledge regarding MPX was developed and adapted based on the available literature. The Chi-square test was used to determine the associations between knowledge levels and independent variables including baseline characteristics. Multivariable logistic regression was also carried out on the significant variables in the bivariate analyses to identify the factors associated with the good knowledge level.

**Results:**

A total of 793 Lebanese adults participated in the study. The overall level of knowledge level regarding human MPX was poor among the Lebanese population; with only 33.04% of them having a good knowledge level ≥ 60%. Knowledge gaps and a substantial poor knowledge level were found in the majority of MPX knowledge domains especially those related to the routes of transmission (76.67%), clinical presentation and symptoms (71.63%), treatment (86.25%), and severity of the disease (91.3%). Interestingly, participants have a good knowledge level of the precautionary measures (80.45%), and the response to a suspected infection (65.20%). Female gender [(aOR = 0.870, CI 95% (0.613–0.941)], increased age 49 [aOR = 0.743, CI 95% (0.381–0.908)], and living in rural areas [aOR = 0.412, CI 95% (0.227–0.861)] were found negatively associated with a good level of knowledge. However, participants with higher educational levels [aOR = 1.243, CI 95% (1.032–3.801)], those working in the medical field [aOR = 1.932, CI 95% (1.331–3.419)], those suffering from chronic disease/immunodeficiency [aOR = 1.231, CI 95% (1.128–2.002)], and participants with moderate/high economic situations [aOR = 2.131, CI 95% (1.431–4.221)] were more likely to have a good knowledge score compared to their counterparts.

**Conclusions:**

The current study pointed out to poor knowledge level regarding MPX among the Lebanese population with substantial knowledge gaps in most aspects of MPX knowledge. The findings stress the urgent need to raise awareness and proactively fill the unveiled gaps, especially among less informed groups.

## Background

Monkeypox (MPX) is a viral zoonotic disease that was first discovered in 1958 among captive cynomolgus macaques, hence the name ‘monkeypox’ [1, 2]. The causative agent of the disease is the monkeypox virus (MPXV), which belongs to the Orthopoxvirus genus within the family Poxviridae [3, 4]. The first recorded human case of MPX was in the Democratic Republic of the Congo (DRC) in 1970 [5]. The disease mainly occurs in Central and West Africa, but the first MPX outbreak outside of Africa was reported in the United States of America (USA) in 2003 [6, 7]. This was linked to contact with infected pet prairie dogs [8]. As a result, people with MPX are occasionally identified outside of endemic countries and are usually associated with imported animals or travel history to endemic areas [6, 9]. Through genome sequencing, two phylogenetically distinct clades of MPXV were identified: the West African clade (WAC) and the Central African (Congo Basin) clade (CAC) [10, 11]. The latter is typically linked to more severe illness, higher mortality, and increased human-to-human transmission [12].

In respect of transmission routes, human-to-human transmission of MPXV was previously thought to mostly occur through respiratory droplets during close and prolonged face-to-face contact by direct contact with body fluids of an ill individual or contact with contaminated objects [13, 14]. However, Ogoina et al. hypothesized that sexual transmission was a possible route of transmission, since sexual intercourse involved close skin-to-skin contact [15, 16]. However, transmission through sexual contact is found to be the main driver of the current outbreaks of HPMX with the majority of cases in men who have sex with men MSM and who have histories indicating a potential exposure during sexual intercourse [17]. To date, it is unknown whether and how the risk of transmission varies with the type of sexual contact and exposure [17, 18]. In addition, less common transmission routes have also been documented, such as mother-to-child transmission or nosocomial infection There is also the hypothetical possibility of transmission through an invasive bite or scratch from an ill animal [18, 21].

The MPX incubation period is typically 6–13 days, although it can also range from 5 to 21 days [22]. The early clinical presentation of HPMX includes a combination of typical symptoms, such fever, headache, chills, tiredness, asthenia, swelling of the lymph nodes, back pain, and muscle aches [23]. Three days following the onset of these prodromal symptoms, a centrifugal maculopapular rash appears, commonly at the site of primary infection, and quickly spreads to other parts of the body [24]. Of note, several factors may affect the severity of the disease, such as the transmission route, host susceptibility, and the quantity of MPXV inoculated [8]. However, most MPX cases experience mild to moderate symptoms [17]. Regarding complications associated with the MPX, encephalitis, dehydration, secondary skin bacterial infections, conjunctivitis, and pneumonia have been reported in endemic countries [25]. The case fatality rate of MPX ranges from 0% to 11% in endemic countries, depending on the clade with mortality disproportionately affecting young children [11]. MPX lethality has been appraised between 3.6% (95% CI (1.7–6.8)] for the West African Clade, and 10.6% [95%CI (8.4–13.3)] for the Central African one [26, 27]. People with compromised immune systems are particularly vulnerable to serious illness [28].

On May 7, 2022, the World Health Organization (WHO) was notified about a confirmed MPX in a traveler who had returned to the United Kingdom from Nigeria [29]. Subsequently, an unprecedented, but not unexpected, outbreak of MPX was recorded in Europe, the Americas, and Australia affecting subjects who had no established travel link to endemic areas [30]. This is the first time that chains of transmission have been reported without known epidemiological links to West or Central Africa [30]. In addition, the rapid spread of MPX has sparked widespread public concern about the eventual evolving of MPX into a global pathogen and its potential to become endemic also in non-African countries [31–33]. It is noteworthy that the ongoing epidemic was associated with the West African lineage of MPX, based on findings of the gene sequencing studies [33]. On July 23, 2022, the WHO declared the current MPX multi-country outbreak, which occurred outside the traditional endemic areas in Africa, a public health emergency of international concern (PHEIC) [34]. This emphasizes the urgent need to raise awareness and issue appropriate guidance for immediate recommended actions, especially in the era of broad social media use, where a flood of misconceptions about MPX are spreading rapidly following the COVID-19 playbook for conspiracy theories.

On June 20, 2022, the first case of MPX was recorded in Lebanon [35]. The investigation revealed that the patient had a travel history to endemic areas. In response, the Ministry of Public Health (MOPH) issued recommendations for the required MPX preventive measures and established a hotline for people to seek information on MPX and report any suspected cases [35]. Although MPX is not highly transmissible and is typically not life-threatening, its emergence underscores the need for a more vigilant disease surveillance system to ensure prompt detection, diagnosis, and effective treatment [36]. As of July 21, 2022, Lebanon has confirmed four cases of MPX linked to travel to endemic areas [37]. Like other countries, Lebanon has not imposed travel-related restrictions or bans on MPX. However, the country has a large diaspora in Africa and is preparing for a busy summer season, with hundreds of thousands of expatriates and tourists expected to visit. As viruses are more likely to spread with increased mobility travel, the risk of MPX importation into Lebanon will also increase. As the situation evolves, and surveillance expands, more cases of MPX are expected to be identified. A growing concern related to the widening MPX outbreak is the possibility of the virus spreading among vulnerable populations, such as children in daycare and schools.

Although Lebanon has reported only four confirmed cases of MPX, it is important for the general population to be knowledgeable and prepared for a possible outbreak [38]. Therefore, raising public health awareness regarding MPX transmission, its risk factors and the required precautionary measures is crucial for self-prevention [39]. However, research from previous viral outbreaks of novel pathogens identified knowledge gaps in transmission and prevention, and the challenges of educating and communicating with the public about an outbreak of an emergent viral pathogen and its associated disease are substantial [40]. As for MPX, a report issued by the WHO revealed that a lack of knowledge about MPX, particularly among healthcare providers, was one of the obstacles in preventing the re-emergence of MPX [41]. Studies conducted in Arab countries have shown a low knowledge level about MPX among Jordanian health students, general practitioners in Indonesia, and physicians in Saudi Arabia [16, 42, 43].

Since knowledge and effective communication about a new virus and associated disease are paramount for reducing morbidity and mortality and helping communities prepare for an outbreak and prevent transmission of the infection [40], it is of great interest to assess the knowledge level of the Lebanese population regarding MPX and unveil knowledge gaps. The relevance of conducting such research in Lebanon is based on the previous evidence of a wide prevalence of conspiracy beliefs and circulating misinformation that was shown in the country during the COVID19 pandemic. In addition, such an assessment can be significant in terms of their preparedness and willingness to protect themselves from a potential infection. Moreover, tackling knowledge can be considered the first step in attempting to change attitudes and behavior. The findings of this study will offer a unique perspective about MPX knowledge levels at the early phase of MPX disease occurrence in Lebanon and could highlight the gaps in knowledge that should be filled, which in turn can orient health communications campaigns.

The overarching goal of the current study was to assess the level of MPX knowledge among the Lebanese adult population and to identify knowledge gaps. It aimed also to identify factors associated with higher levels of MPX knowledge among this population.

## Materials and methods

### Study design

This paper is part of a large project assessing the knowledge, attitudes, risk perceptions, and beliefs of the Lebanese population regarding monkeypox. The survey was conducted during the early phase of the MPX outbreak which coincided with the recording of the first cases of human MPX in the country. An observational online cross-sectional survey was carried out over the first 2 weeks of August 2022, following the STROBE (Strengthening the reporting of observational studies in epidemiology) [44]. The study involvied Lebanese adults recruited from all Lebanese provinces (Akkar, North, Bekaa, Baalbeck-Hermel, South, Nabatieh, Beirut, Mount Lebanon). Lebanese adults aged 18 years or older who can speak, read and understand the Arabic language and those who have internet literacy and access were eligible to participate. The study excluded non-Lebanese adults living in Lebanon, Lebanese adults who were not willing to read and understand the Arabic language, illiterate adults, those who were living abroad at the time of the study, and those who lacked internet literacy. Adults who refused to participate were also excluded. As there were no previous studies in Lebanon about knowledge, attitudes and practices (KAP) towards MPX and in light of the rapidly evolving nature of the disease and the need to fill the gaps in knowledge and develop rapid measures to contain the spread of the virus, a convenience sampling method was used to recruit participants from all regions in Lebanon.

### Questionnaire development

An extensive review of the literature was conducted to identify relevant items on MPX knowledge, attitude, and recommended preventive measures among the population [16, 33, 42, 45, 46]. A self-reported, structured questionnaire was initially developed in English and designed by the authors to cover broad aspects of MPX knowledge (etiology, clinical presentation, severity, transmission routes, case management, vaccines, and precautionary measures) among the population. The content validity of the questionnaire was assessed by a panel of experts using qualitative and quantitative methods. Items were assessed for the clarity and relevance of the domains. The reliability of the English knowledge scale was evaluated using Cronbach alpha (α = 0.88 ≥ 0.70) [47, 48]. Then, the original English draft of the questionnaire was translated and adapted to the Arabic language based on the standard translation guidelines. Two independent certified bilingual translators performed the initial translation. Any inconsistencies were discussed and resolved. The translated version was then back-translated into English by two native English speakers. A committee of experts was formed to review the translated version for linguistic issues, problematic items, and discrepancies in wording and ambiguity. The committee’s goal was to ensure authenticity and to reach consensus on any ambiguous terminology. After reviewing all items, a consensus was reached to keep all items, resulting in the pre-final version of the translated questionnaire. Then, the reliability and the content validity of the Arabic version of the questionnaire were also checked. The Arabic version revealed good reliability (α = 0.91 ≥ 0.70) and validity (CVI > 0.8) [49, 50]. Test–retest reliability was also evaluated through a survey on 20 Lebanese adults completing the questionnaire at two different points in time (22 and 28 July 2022). Items whose corresponding correlation coefficient in T1 vs. T2 was > 0.80 were considered “consistent”, and were ultimately included in the final questionnaire that was then delivered by 10 August 2022. The questionnaire was pre-tested among 30 Lebanese subjects for assessing survey flow, functionality, readability, comprehension of instructions, and clarity. Based upon feedback from the pre-test, minor modifications in terms of readability and clarity were made to the questionnaire. The questionnaire’s reliability was also tested, and the Cronbach Alpha value was 0.89. The average time for completing the survey was 13 min.

The questionnaire was self-administered, and its final version consisted of open-ended questions and was divided into two main sections:

**The baseline characteristics of the study participants section** included information about age, gender, marital status, urbanicity, occupation, and education level of the participants. In addition to socio-demographic characteristics, this section also included an item on self-reported general health status (“In general, how would you rate your state of health?) and an item related to immunodeficiency and chronic illness (“Do you suffer from an immunodeficiency or chronic, that is to say long-lasting, disease or health problem that requires medical attention (for example, diabetes, HIV, heart or respiratory disease?). Participants were asked to disregard temporary or temporary health problems, such as the flu. Specific items regarding the pandemic included whether participants had been diagnosed with MPX and whether some of their relatives/acquaintances (family members, friends, or neighborhood) had been infected were also queried. Participants were also asked to rank their economic situation.

**Knowledge about MPX** consisted of 55 questions assessing awareness of the Lebanese population about different aspects of MPX knowledge. This section included seven domains:

- **The general knowledge domain** consisted of nine questions addressing adults' knowledge about the etiology of the disease, geographical areas affected by the virus, incubation period, and period of communicability [16, 51, 52].
- **The routes of the transmission domain** included seven items targeting possible transmission routes (respiratory droplets, close physical skin-to-skin contact, close contact with infectious material from skin lesions of an infected person, vertical transmission, and sexually transmitted infection…) [51–54].
- **The clinical presentation of the disease domain** included 10 questions about the symptoms of the disease [51, 52, 55, 56].
- **The severity domain** consisted of eight questions assessing participants' knowledge about the seriousness of MPX, lethality, and potential complications of the disease [17, 51, 52].
- **The case management domain** comprised nine questions linked to the availability of a specific treatment, the availability of a vaccine against MPX, the possibility of using antibiotics to treat MPX, the isolation of the patient, and supportive treatment [25, 51, 52, 56].
- **The precautionary measures domain** included seven questions assessing participants’ knowledge about required precautionary measures to protect themselves from the infection (physical distancing, hand hygiene, wearing a facemask…) [51, 52, 57].
- **The appropriate response to the MPX exposure domain** consisted of five questions that pinpointed the adult’s appropriate knowledge about what they should do in case of exposure to MPX case or suspicion of being infected such as self-isolation and avoiding physical contact with others, notification of health authorities, monitoring of symptoms, notification of close contacts about possible exposure and seeking care [51, 52].

All the items were answered on a true/false basis and an additional “do not know” option. A correct response had a value of ‘1’ and a “wrong” or do not know response had a value of ‘0’. Hence, the aggregate overall score for all 55 knowledge questions would range from 0 to 55 points. Participants ‘overall knowledge was categorized using modified Bloom’s cutoff point, as good if the score was ≥ 60% (33–55 points), and poor if the score was less than 60% (< 33 points) [58]. Internal consistency of the knowledge sections was measured through calculation of the Cronbach’s alpha. Cronbach’s alpha is a measure of how closely related a set of items are as a group and is usually considered a scale of reliability. In general, a score ≥ 0.7 is considered acceptable. Subscales were computed and categorized using the same abovementioned methodology. Of note, Cronbach alpha was ≥ 0.70 for all of the subscales showing good reliability [49, 50].

Participants were also asked about their sources of information regarding HPMX and to rank the reliability of these sources.

### Sample size calculation

The Raosoft sample size calculator designed specifically for population surveys was used to calculate the required sample size [59]. Since no previous study in Lebanon examined the population’s knowledge of MPX, a conservative estimate of 50% was used. An estimated sample size of 383 was calculated based on a 95% confidence level, and an absolute error of 5%. To reduce the sampling error and to increase the study power, a rough estimation was made by multiplying the calculated sample size by 2 times, leading to a final sample size of 793 participants.

### Ethical considerations

The research protocol was properly reviewed and approved by the ethical committee at Rafic Hariri University Hospital (RHUH) (reference number 2022-0801). All methods were performed following the ethical standards as laid down in the Declaration of Helsinki and its later amendments. Informed electronic consent will be obtained from the respondents using the following item in the introductory section of the survey: “Do you agree to participate in this study?”. The respondent had to respond “Yes” to be able to participate in this study. There are no known direct benefits associated with participating in this research study. Participation in this study is voluntary and participants may refuse to participate or withdraw at any point during the survey. However, when finalizing the survey, participants will be unable to withdraw their data. Participants will receive no monetary compensation or gifts for participating in this study.

### Data collection

The online survey was created in Arabic language using Google Forms. The questionnaire was shared through social media platforms, including Facebook, Instagram, and LinkedIn, using the accounts of the research team members. In addition, a Facebook page was created, and the study was boosted on different social media platforms such as Facebook and Instagram to ensure a large-scale distribution and recruitment of participants. Furthermore, the research team members shared the survey link through email lists and the chatting group “WhatsApp.” Reminders were also sent to the contacts to encourage them to fill out the survey. The survey link was estimated to have been sent to approximately 17,000 individuals. In some cases, participants were asked to share the link to the study with their acquaintances on their social media accounts. As the total number of individuals who received the questionnaire is unknown, it is impossible to calculate the response rate for this study. The link included a summary of the study's purpose and instructions on how to fill out the questionnaire. The first page of the questionnaire included a set of dichotomous questions to screen for eligibility and digital informed consent. Participants were then asked about their consent for study participation through a specific dichotomous question (i.e., Yes vs. No). To prevent participants from looking up the correct responses, a clear statement was provided at the beginning of the survey, asking participants to respond to the questions based on their current knowledge. The survey was anonymous, and no personal data such as name, IP address, email address, or any other unnecessary personal information was requested, saved, or tracked. Although no monetary compensation was offered to the participants, they were guaranteed that a full explanation of all items would be provided at the end of the questionnaire, representing an educational opportunity on MPX. To avoid missing data, a response to all items was mandatory. Although anyone who received the survey and agreed to participate was able to fill out the survey, only respondents who accurately completed the questionnaire and met the eligibility criteria were considered for subsequent analyses.

### Data analysis

All the statistical analyses were performed using the statistical software SPSS (Statistical Package for Social Sciences), version 22.0. The normality of the distribution of knowledge scale variables was checked using the Kolmogorov–Smirnov test (K–S). Categorical variables were reported by frequencies and percentages. The Chi-square test was used to determine the associations between knowledge levels and independent variables including baseline characteristics. Multivariable logistic regression was also carried out on the significant variables in the bivariate analyses/chi-squared test with a *p* value < 0.2 to identify the factors associated with the good level of knowledge among the Lebanese population. For all tests, *p* values < 0.05 were considered statistically significant.

## Results

### Characteristics of the study sample

A total of 793 Lebanese adults who met the inclusion criteria completed the survey. The distribution of survey participants across age, geographical areas, and health-related variables suggests that a national sample was achieved. Table 1 displays the baseline characteristics of the participants. The majority of surveyed adults were females (70.87%), married (57.63%), aged less than 30 years (44.64%), and had an advanced educational level (75.66%). Most of them resided in urban areas (74.15%), particularly in Mount Lebanon province (30.52%). Less than 30% of them worked in the medical field. The majority of participants rated their health status as good or above (74.91%), and only 19.29% reported suffering from chronic diseases or immunodeficiency. Most of them (77.56%) had a low economic status. Only 9.37% of participants had heard about MPX before the current multicountry outbreak, and only 0.50% reported previous knowledge of someone contracting HPMX. Notably, none of them reported a previous history of HPMX.

**Table 1:** Socio-demographics characteristics of the participants (*N* = 793)

	*n*	%
Gender		
Male	231	29.13
Female	562	70.87
Age groups (years)		
18–29 years	354	44.64
30–49 years	249	31.40
50 years and above	190	23.96
Marital status		
Single	277	34.93
Married/Engaged	457	57.63
Other (divorced/widowed)	59	7.44
Education level		
Secondary or below	193	24.34
University or above	600	75.66
Occupation		
Outside the medical field	389	49.05
Medical field	219	27.62
Not working	185	23.33
Urbanicity		
Urban	588	74.15
Rural	205	25.85
Province		
Great Bekaa	118	14.90
Great North (Akkar/North)	96	12.10
South and Nabatieh	109	13.70
Beirut	228	28.75
Mount Lebanon	242	30.52
Self-reported health status		
Fair or below	199	25.09
Good or above	594	74.91
Self-reported economic status		
Low	615	77.56
Moderate/high	178	22.44
Suffering from an immunodeficiency or chronic disease		
No	640	80.71
Yes	153	19.29
Heard before about MPX (before the current outbreak)		
No	743	90.63
Yes	50	9.37
Knowing someone with MPX		
No	789	99.50
Yes	4	0.50

*N* frequency, %: percentage

### Lebanese adults’ knowledge about MPX

#### General knowledge about MPX

**Fig. 1:**
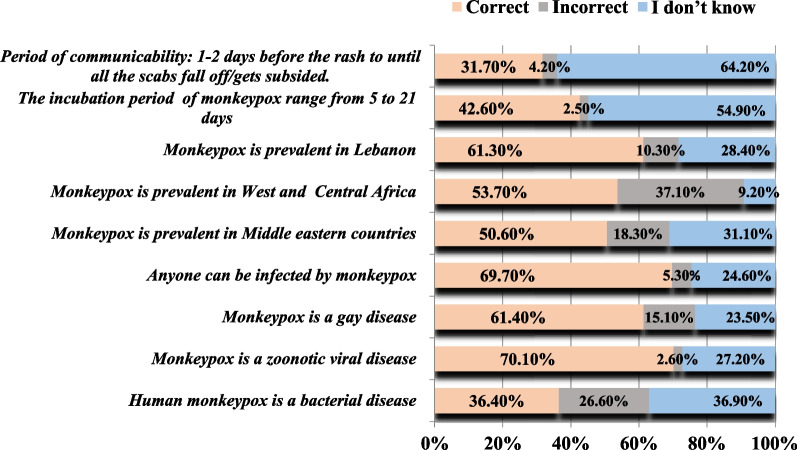
General knowledge of Lebanese adults regarding MPX

Briefly, the majority of Lebanese adults acknowledged MPX as a zoonotic viral disease (70.1%) that can affect anyone (69.7%) and is not only restricted to gay individuals (61.4%). Moreover, around half of the participants (53.7%) were aware that MPX is only prevalent in West and Central Africa and not in Middle Eastern countries (50.6%), including Lebanon (61.3%). Focusing on the incubation period and communicability, only 42.6% of Lebanese adults were cognizant that the incubation period of MPX ranges from 5 to 21 days. Furthermore, the period of communicability was only acknowledged by 31.7% of the participants (Fig. 1). It is worth mentioning that there is a high proportion of uncertainty among participants regarding the question. This was revealed by the “I don’t know” answer to the majority of questions compared to “incorrect” answers. The most significant uncertainty among the respondents was represented by the inappropriate understanding of the communicability and the incubation period. This suggests that Lebanese adults acknowledge their lack of knowledge regarding the disease.

#### Knowledge regarding routes of transmission of MPX

**Fig. 2:**
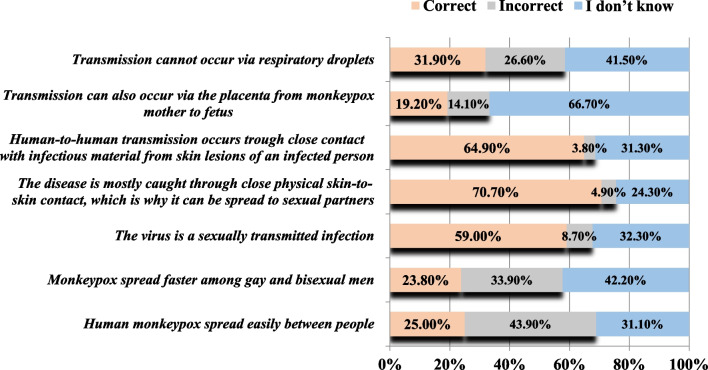
Knowledge of the Lebanese adults regarding transmission routes of MPX

The knowledge gaps were particularly evident in the domain of MPX routes of transmission, as the majority of participants were unable to identify potential transmission modes of MPX. Notably, only 25% of participants were aware that MPX does not spread easily between people. Only 59.0% of participants were aware that MPX is not a sexually transmitted disease. Interestingly, the majority of participants (70.7%) acknowledged the potential transmission of MPX through close physical skin-to-skin contact, which is why it can be spread to sexual partners, as well as through close contact with infectious material from skin lesions of an infected person (64.9%). On the contrary, only 19.2% were aware that transmission can also occur via the placenta from an MPX-infected mother to her fetus. Another plausible route of transmission, such as respiratory droplets, was acknowledged by only 31.9% of the participants. A similar pattern of uncertainty was also evident in this domain of knowledge about transmission routes (Fig. 2). The most significant area of uncertainty among the respondents was the transmission of MPX via the placenta from an infected mother to her fetus (66.7%).

#### Knowledge regarding clinical presentation of MPX

**Fig. 3:**
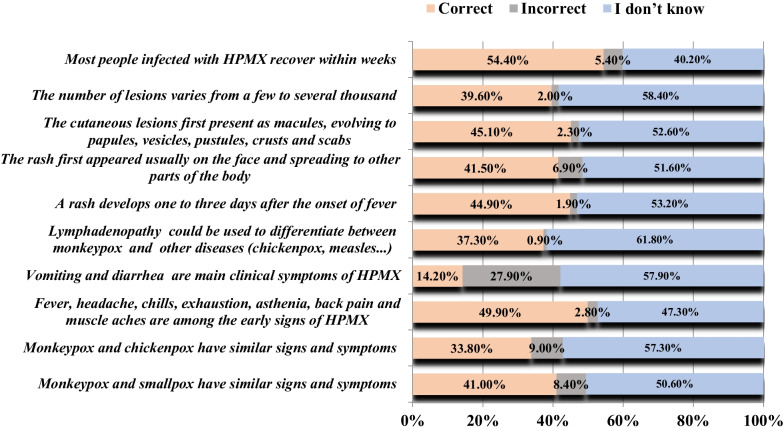
Knowledge of Lebanese adults regarding clinical presentation of MPX

Focusing on the clinical presentation of MPX, less than half of the participants were aware that MPX and smallpox have similar signs and symptoms. Only half of the participants were aware of the early signs of MPX (fever, headache, chills, exhaustion, asthenia…) and that recovery of most people infected with MPX occurs within weeks. Notably, only 14.2% of the respondents admit that vomiting and diarrhea are not the main clinical symptoms of MPX. As for rash and lesions, less than 40% of participants were aware of the date of onset of rash, their distribution in the body, and the number of lesions. In addition, the majority of respondents did not recognize that lymphadenopathy is one clinical that could be used to differentiate between MPX and other diseases. This latter was expected among the population (Fig. 3).

#### Knowledge regarding the severity of MPX

In terms of high-risk groups, the participants were not able to identify those at high-risk of MPX. Only one-quarter of them were aware that elderly people are not a high-risk group for MPX, and only 52.1% identified healthcare workers as high risk. In addition, the majority were not aware that the clinical manifestation of MPX is usually not severe, and that this disease is less contagious than smallpox and even causes less severe illnesses. Only half of them knew that MPX is not a deadly disease (50.2%). However, the majority recognized that the occurrence of complications depends on the extent of virus exposure and the patient's current health status (69.5%), and that children and people with immunodeficiency (66.6%) were more likely to suffer from complications. Notably, corneal involvement (which may lead to loss of vision) was not recognized by the majority of participants as a possible complication of HPMX (Table 2).

**Table 2:** Answers of the surveyed Lebanese adults regarding HPMX severity, case management, vaccines, and precautionary measures

	Correct	Incorrect	I do not know
	*n* (%)	*n* (%)	*n* (%)
Severity of the disease and high-risk groups			
The elderly were at high risk of HPMX^f^	205 (25.9%)	149 (18.8%)	439 (55.4%)
Healthcare workers were at high risk of HPMX^c^	413 (52.1%)	111 (14.0%)	269 (33.9%)
HPMX is less contagious than smallpox and causes less severe illnesses^c^	116 (14.6%)	187 (23.6%)	490 (61.8%)
The clinical manifestation of HPMX is usually severe^f^	89 (11.2%)	231 (29.1%)	473 (59.6%)
HPMX is a deadly disease^f^	398 (50.2%)	84 (10.6%)	311 (39.2%)
Complications due to HPMX can occur among children/people with immunodeficiency^c^	528 (66.6%)	0 (0%)	265 (33.4%)
Complications are related to the extent of virus exposure and patient health status^c^	551 (69.5%)	11 (1.4%)	231 (29.1%)
Corneal involvement (leading to loss of vision) is one of the complications of HPMX^c^	156 (19.7%)	142 (17.9%)	495 (62.4%)
Treatment, vaccines, and case management			
There is no specific treatment for HPMX^c^	454 (57.3%)	32 (4.0%)	307 (38.7%)
Patients should be offered fluids and food to maintain adequate nutritional status^c^	557 (70.2%)	22 (2.8%)	214 (27.0%)
The smallpox vaccine can provide cross-protection for the HPMX virus^c^	337 (42.5%)	116 (14.6%)	340 (42.9%)
There is a specific vaccine for HPMX^f^	108 (13.6%)	253 (31.9%)	432 (54.5%)
The isolation of the HPMX patient in the hospital/at home in a separate room is mandatory^c^	544 (68.6%)	9 (1.1%)	240 (30.3%)
Covering skin lesions (e.g., long sleeves, long pants) is not necessary for HPMX cases ^f^	143 (18.0%)	237 (29.9%)	413 (52.1%)
Isolation should be continued until the fever disappear^f^	43 (5.4%)	405 (51.1%)	345 (43.5%)
Patients with HPMX can receive antibiotics that alleviate symptoms^f^	116 (14.6%)	196 (24.7%)	481 (60.7%)
It is not necessary to closely monitor the HPMX case during the period of isolation^f^	375 (47.3%)	158 (19.9%)	260 (32.8%)
Precautionary measures			
Avoid contact with any materials that have been in contact with a suspected case of HPMX^c^	729 (91.9%)	5 (0.6%)	59 (7.4%)
Keep safe physical distance^c^	641 (80.8%)	13 (1.6%)	139 (17.5%)
Do not touch the rash or scabs of a person with HPMX^c^	741 (93.4%)	0 (0%)	52 (6.6%)
Do not kiss, hug, cuddle or have sex with someone with HPMX^c^	750 (94.6%)	1 (0.1%)	42 (5.3%)
Wear a face mask if you are in close contact with someone who has symptoms^c^	546 (68.9%)	100 (12.6%)	147 (18.5%)
Eating raw meat^f^	461 (58.1%)	89 (11.2%)	243 (30.6%)
Practice good hand hygiene^c^	660 (83.2%)	10 (1.3%)	123 (15.5%)

*N* frequency, %: percentage, ^c^refers to a correct statement, ^f^refers to a false statement

#### Knowledge regarding vaccines and case management:

Approximately half of the surveyed adults were aware that there is no specific treatment available for HPMX. Interestingly, the majority of participants recognized that supportive treatment is necessary, including providing fluids and food to maintain proper nutritional status (70.2%). On the other hand, only 13.6% of participants were aware of the existence of a specific vaccine for MPX. In addition, only 14.6% acknowledged that antibiotics cannot be used to treat MPX and alleviate symptoms. The cross-protection offered by the smallpox vaccine was recognized by 42.5% of participants. Although the majority of participants (68.6%) knew that MPX cases need to be isolated in a separate room (either at home or in the hospital), only 47.3% were aware of the need to closely monitor MPX cases during this period. Notably, over 90% of surveyed adults lacked knowledge about the duration of isolation (see Table 2).

#### Knowledge about precautionary measures

Interestingly, the majority of participants (> 80%) were knowledgeable about the different precautionary measures that should be adopted to protect themselves against MPX including keeping a safe physical distance (80.8%), practicing good hand hygiene (83.2%), avoiding contact with any materials of a suspected case (91.9%) or touching the rash or scabs of MPX case (93.4%), wearing facemask (68.9%) and avoiding eating raw meat (58.1%) (Table 2).

#### Knowledge regarding response to possible exposure to MPX

**Fig. 4:**
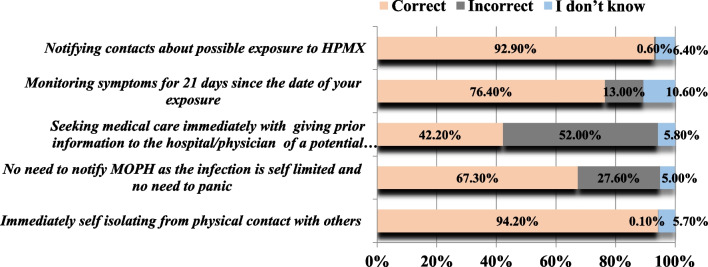
Knowledge of Lebanese adults regarding response to possible exposure to MPX

Almost all of the participants were aware of the necessity to self-isolate immediately in case of suspecting or catching MPX (94.2%) and to notify their close contacts about their potential exposure (92.9%). More than half of the surveyed adults were cognizant of the requirement to notify the health authorities about any suspicion of contracting the disease or possible exposure, even if the infection is self-limited. Around the third-quarters of participants were knowledgeable that symptoms should be monitored for 21 days since the date of exposure. Even though most of the participants (61%) were able to identify the appropriate response to possible exposure to MPX, only 42.2% of them acknowledged the need to provide prior information to the hospital/physician about a potential infection before seeking medical care (Fig. 4).

#### Overall knowledge level and specific knowledge domains

**Fig. 5:**
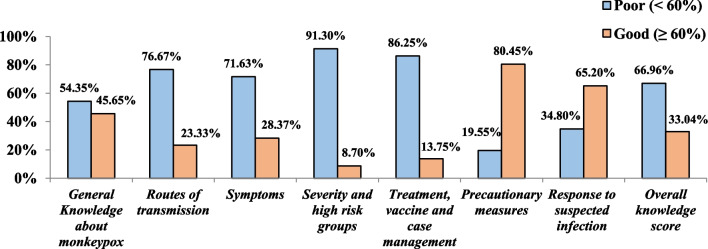
Knowledge levels of the Lebanese population about MPX per domain

The overall level of knowledge regarding human MPX was poor among the majority of the Lebanese population, with only 33.04% having a good knowledge level of ≥ 60% (Fig. 5). All domains of knowledge in the construct were found to be reliable with Cronbach alpha as follows: general knowledge (α = 0.78), routes of transmission (α = 0.82), clinical presentation of the disease (α = 0.87), severity of the disease (α = 0.74), case management (α = 0.91), precautionary measures (α = 0.93), and appropriate response to MPX exposure (α = 0.79). Sustainable knowledge gaps were revealed in the different domains of MPX knowledge. Notably, more than half of the participants (54.35%) had a good knowledge level in the general knowledge domain. However, the majority of participants had a poor knowledge level in the following domains: routes of transmission (76.67%), clinical presentation and symptoms (71.63%), treatment (86.25%), and severity of the disease (91.3%). On the contrary, 80.45% of participants had a good knowledge level in the precautionary measures’ domain, and 65.20% of them were knowledgeable about the appropriate response to a suspected infection (Fig. 5).

### Sources of information and knowledge level

As shown in Table 3, the main sources of information that the Lebanese population used to get information about MPX were media (68.72%) followed by health authorities (61.03%), and social media (58.38%). The hotline dedicated by the MOPH was the least source of information used (1%). A significant relationship was found between knowledge score and sources of information about monkeypox (*p* < 0.001). The majority of participants with a good knowledge level reported scientific journals/research articles (42.1%), health websites (38.9%), health authorities (38.4%), and healthcare providers (36.9%) as their information sources compared to those who used TV and radio (32.3%), social media (30%), and family or friends (28.3%).

**Table 3:** Relationship between knowledge score and sources of information about monkeypox

	Overall knowledge			*P* value
	Total	Poor	Good	
	*n* (%)	*n* (%)	*n* (%)	
Media (TV, radio, newspaper)	545 (68.73%)	369 (67.7%)	176 (32.3%)	< 0.001
Social media (Whatsapp, Facebook, Twitter…)	463 (58.39%)	324 (70%)	139 (30%)	
Health authorities (MOPH…)	484 (61.03%)	298 (61.6%)	186 (38.4%)	
Healthcare professionals (physicians…)	328 (41.36%)	207 (63.1%)	121 (36.9%)	
Scientific journals/articles	420 (52.96%)	243 (57.9%)	177 (42.1%)	
Health website (WHO, CDC…)	422 (53.22%)	258 (61.1%)	164 (38.9%)	
Family and friends	350 (44.14%)	251 (71.7%)	99 (28.3%)	
Hotline	8 (1%)	5(62.5%)	3 (37.5%)	

#### Factors associated with the good overall knowledge level

A good level of knowledge was found to be negatively associated with female gender, increased age, and living in rural areas. Female participants were more likely to have a poor knowledge level compared to males [aOR = 0.870, CI 95% (0.613–0.941)]. Increased age was negatively associated with a good level of knowledge; participants aged between 30 and 49 [aOR = 0.743, CI 95% (0.381–0.908)] and those aged 50 and above [aOR = 0.514, CI 95% (0.317–0.771)] were less likely to have a good knowledge level compared to young participants aged between 18 and 29 years. Participants living in rural areas [aOR = 0.412, CI 95% (0.227–0.861)] were less likely to have a good knowledge score compared to those living in urban areas. However, participants with a higher educational level [aOR = 1.243, CI 95% (1.032–3.801)], those working in the medical field [aOR = 1.932, CI 95% (1.331–3.419)], those suffering from chronic disease/immunodeficiency [aOR = 1.231, CI 95% (1.128–2.02)], and participants with a moderate/high economic situation [aOR = 2.131, CI 95% (1.431–4.221)] were more likely to have a good knowledge score compared to their counterparts (Table 4).

**Table 4:** Multivariable analysis of the factors associated with the knowledge score

	Overall knowledge score						
	Poor	Good	Total	*P* value	aOR	CI 95%	
	*n* (%)	*n* (%)	*n* (%)			Lower	Upper
Gender				**0.032**			
Male	138 (59.7%)	93 (40.3%)	231 (100%)	Ref.			
Female	393 (69.9%)	169 (30.1%)	562 (100%)		0.870	0.613	0.941
Age (years)				**0.003**			
18–29	230 (65.0%)	124 (35.0%)	354 (100%)	Ref.			
30–49	159 (63.9%)	90 (36.1%)	249 (100%)		0.743	0.381	0.908
50 and above	142 (74.7%)	48 (25.3%)	190 (100%)		0.514	0.317	0.771
Marital status				0.391			
Single	181 (65.3%)	96 (34.7%)	277 (100%)				
Married	296 (64.8%)	161 (35.2%)	457 (100%)				
Other (widowed/divorced)	54 (91.5%)	5 (8.5%)	59 (100%)				
Educational level				**0.048**			
Secondary or below	120 (62.8%)	73 (37.2%)	193 (100%)	Ref.			
University or above	396 (66.0%)	204 (34.0%)	600 (100%)		1.243	1.032	3.801
Occupation				**0.006**			
Outside the medical field	281 (72.2%)	108 (27.8%)	389 (100%)	Ref.			
Medical field	129 (58.9%)	90 (41.1%)	219 (100%)		1.932	1.311	3.419
Not working	121 (65.4%)	64 (34.6%)	185 (100%)		1.132	0.821	2.712
Urbanicity				**0.023**			
Urban	348 (59.2%)	240 (40.8%)	588 (100%)	Ref.			
Rural	183 (89.3%)	22 (10.7%)	205 (100%)		0.412	0.227	0.861
Suffering from chronic disease/immunodeficiency				**0.032**			
No	438 (68.4%)	202 (31.6%)	640 (100%)	Ref.			
Yes	93 (60.8%)	60 (39.2%)	153 (100%)		1.231	1.128	2.002
Self-reported economic situation				**< 0.001**			
Low	499 (81.1%)	116 (29.9%)	615 (100%)	Ref.			
Moderate/high	100 (56.2%)	78 (43.8%)	178 (100%)		2.131	1.431	4.221

*N* frequency, *%* Percentage, *aOR* adjusted odds ratio, *CI* confidence interval

#### Factors associated with each knowledge domain

Participants with higher levels of education and those working in the medical field were more likely to have a good general knowledge level compared to their counterparts. Regarding good knowledge levels on the transmission routes of MPX, female participants, those aged 50 years and above, and those living in rural areas were less likely to report a good knowledge level. However, surveyed adults who worked in the medical field, had high levels of education, suffered from chronic diseases or immunodeficiency, and had moderate to high economic situations were more likely to report a good knowledge level. These factors were also found to be associated in the same direction with the severity of the MPX knowledge domain and the clinical presentation of MPX (except for age). Knowledge in the treatment and case management domains was negatively associated with the female gender and being residents of rural areas. On the other hand, it was positively associated with higher levels of education and socioeconomic status as well as being engaged in the medical field. Lastly, a good level of knowledge regarding an appropriate response to an exposure or a suspicion of MPX was found to be positively associated with being involved in the medical field and negatively associated with rural residence (Table 5).

**Table 5:** Factors associated with each knowledge domain

	General knowledge			
	*P* value	aOR	CI 95%	
			Lower	Upper
Educational level	0.003			
Secondary or below	Ref.			
University or above		1.128	1.019	2.714
Occupation	0.046			
Outside the medical field	Ref.			
Medical field		1.378	1.211	2.828
Not working		1.042	0.819	4.322
Transmission routes				
Gender	< 0.001			
Male	Ref.			
Female		0.870	0.613	0.941
Age (years)	< 0.001			
18–29	Ref.			
30–49		1.233	0.722	2.104
50 and above		0.364	0.122	0.563
Educational level	< 0.001			
Secondary or below	Ref.			
University or above		1.243	1.032	3.801
Occupation	< 0.001			
Outside the medical field	Ref.			
Medical field		2.923	1.770	4.828
Not working		1.912	0.733	2.512
Urbanicity	< 0.001			
Urban	Ref.			
Rural		0.293	0.174	0.492
Suffering from chronic disease/immunodeficiency	0.032			
No	Ref.			
Yes		1.151	1.072	1.318
Self-reported economic situation	< 0.001			
Low	Ref.			
Moderate/high		2.441	1.397	4.183
Clinical presentation of the MPX				
Gender	0.032			
Male	Ref.			
Female		0.870	0.613	0.941
Educational level	0.048			
Secondary or below	Ref.			
University or above		1.243	1.032	3.801
Occupation	0.007			
Outside the medical field	Ref.			
Medical field		3.237	1.410	7.435
Not working		1.216	0.482	3.067
Urbanicity	0.003			
Urban	Ref.			
Rural		0.812	0.427	0.942
Suffering from chronic disease/immunodeficiency	0.007			
No	Ref.			
Yes		1.204	1.137	4.146
Self-reported economic situation	< 0.001			
Low	Ref.			
Moderate/high		1.883	1.321	3.122
Severity of MPX				
Gender	0.032			
Male	Ref.			
Female		0.870	0.613	0.941
Age (years)	0.003			
18–29	Ref.			
30–49		0.743	0.381	0.908
50 and above		0.514	0.317	0.771
Educational level	0.048			
Secondary or below	Ref.			
University or above		1.243	1.032	3.801
Occupation	0.006			
Outside the medical field	Ref.			
Medical field		1.932	1.311	3.419
Not working		1.132	0.821	2.712
Urbanicity	0.023			
Urban	Ref.			
Rural		0.412	0.227	0.861
Suffering from chronic disease/immunodeficiency	0.032			
No	Ref.			
Yes		1.231	1.128	2.002
Self-reported economic situation	< 0.001			
Low	Ref.			
Moderate/high		1.881	1.492	5.034
Treatment, vaccines, and case management				
Gender	0.002			
Male	Ref.			
Female		0.717	0.489	0.894
Educational level	0.011			
Secondary or below	Ref.			
University or above		2.328	1.417	5.043
Occupation	< 0.001			
Outside the medical field	Ref.			
Medical field		2.132	1.422	4.129
Not working		1.782	0.773	4.237
Urbanicity	< 0.001			
Urban	Ref.			
Rural		0.741	0.539	0.948
Self-reported economic situation	< 0.001			
Low	Ref.			
Moderate/high		1.749	1.138	3.918
Precautions measures				
Age (years)	< 0.001			
18–29	Ref.			
30–49		0.858	0.461	1.596
50 and above		0.124	0.040	0.386
Occupation	0.024			
Outside the medical field	Ref.			
Medical field		1.833	1.083	3.103
Not working		1.152	0.639	2.077
Urbanicity	0.002			
Urban	Ref.			
Rural		0.362	0.191	0.688
Suffering from chronic disease/immunodeficiency	0.032			
No	Ref.			
Yes		1.913	1.098	3.334
Self-reported economic situation	< 0.001			
Low	Ref.			
Moderate/high		1.749	1.138	3.918
Response to possible exposure				
Occupation	0.002			
Outside the medical field	Ref.			
Not working		1.588	0.926	3.780
Urbanicity	0.018			
Urban	Ref.			
Rural		0.644	0.382	0.879

*aOR* adjusted odds ratio, *CI* confidence interval

## Discussion

Good knowledge about the MPX virus and associated disease is paramount for helping communities to better prepare and properly respond to a possible MPX outbreak. Lack of awareness can hinder the community’s ability to prevent and control the transmission of the infection. Therefore, it is important that the Lebanese population has a sufficient understanding of MPX, including early identification, transmission routes, and preventive measures. Until now, there has been no information on the level of knowledge about MPX in the Lebanese community. Thus, this study provides an overall view of public awareness of MPX and highlights knowledge gaps in the early phase of MPX in Lebanon. Identifying knowledge gaps is crucial to implementing preventive measures, especially primary prevention, and to guide communication campaigns to achieve better outcomes in terms of preparedness and reducing morbidity and mortality. The study also explores factors that influence knowledge about MPX.

### Main findings

The main finding of this study was that the majority (66.96%) of the Lebanese population had a poor level of knowledge regarding MPX. Significant knowledge gaps and uncertainties were revealed in most of the MPX knowledge domains, particularly those related to transmission routes, clinical presentation, treatment, and disease severity. On the other hand, participants exhibited a good level of knowledge regarding precautionary measures and how to respond to suspected infection. A poor level of knowledge was associated with female gender, increased age, and living in rural areas, while higher education levels, working in the medical field, having chronic diseases/immunodeficiency, and moderate/high economic situations were associated with a good knowledge score compared to their counterparts.

Some explanations for the gaps and uncertainties in the knowledge of MPX are quite straightforward. First, MPX is neither endemic in Lebanon nor its neighboring countries. The recency of the disease, not in terms of its origin but rather in terms of its appearance in non-endemic locations, could explain the poor knowledge level revealed in this study. Given the rarity of MPX cases in the country (only four sporadic travel-related cases), these findings in terms of poor knowledge level were expected. In fact, the Lebanese population is not familiar with this infection, since the disease originates from central Africa and usually spreads in the tropical rainforest region. Second, the majority of the participants were aged less than 50 years; therefore, they were born in a “Variola-free” world and did not have previous information regarding the disease. Another explanation is that the Lebanese population is currently experiencing a multi-layered, overlapping shock that led the country into a nationwide socio-economic crisis. In addition, the majority of Lebanese people (82%) live in multidimensional poverty, which takes into account factors other than income, such as access to health, education, and public utilities (Multidimensional Poverty in Lebanon: Painful Reality and Uncertain Prospects).

In this regard, several studies have highlighted a potential link between low knowledge levels on diseases and low economic situations. Although there is no doubt that the resilience of the Lebanese people has been demonstrated again in these crises, the truth is that we are facing an exhausted population in one of the most complicated years since the civil war. People are overwhelmed by the need to preserve their basic needs as well as their access to essential healthcare and medications. This shift in priorities could restrain their proactivity in seeking information about a potential disease, such as MPX.

Lastly, it should be mentioned that the coverage of this topic by local media has been sluggish and timid compared to what was shown during the COVID-19 pandemic. However, such a dearth of knowledge concerning transmission, treatment, and symptoms can negatively affect the control of the disease. Exotic agents are a threat to public health systems due to limited experience in case management and a lack of appropriate resources. Therefore, raising the population’s level of knowledge and awareness is suggested to prevent the disease from spreading across the world successfully.

Interestingly, a common aspect of poor knowledge and uncertainties has been found in several studies conducted previously about uncommon outbreaks in the study areas [60–62]. For example, a number of studies conducted in Indonesia revealed that healthcare workers had high knowledge about Indonesia’s endemic outbreaks and a low level of knowledge regarding other rare outbreaks. In regard to MPX, a similar pattern of uncertainty and dearth of knowledge was reported in recent studies that tackled MPX knowledge. Either among the general population or among healthcare workers which aligns with our findings. A study conducted among the general population in Saudi Arabia showed that more than half (52%) of participants had low knowledge about MPX infection. Another study conducted in Iraq showed that most of the surveyed subjects had insufficient knowledge of this infection. Although healthcare workers and medical students are supposed to be more knowledgeable about this subject, several knowledge gaps were revealed. For example, a study conducted in Indonesia showed that only 10% of general practitioners could correctly answer 80% of questions about monkeypox [45]. Another recent study conducted in Italy found that medical professionals’ knowledge of monkeypox was relatively unsatisfactory, with significant knowledge gaps in this subject [33]. Similar results were found in a recent study conducted among medical students Jordanian medical, nursing, dentistry, and pharmacy students, and Saudi physicians [16, 42].

It is noteworthy that less than 10% of the adults surveyed reported having heard of MPX prior to the ongoing outbreak. This result could affect our positive findings in terms of good knowledge in the precautionary measures and response domains. Participants may have chosen these measures based on their previous experience with COVID-19, rather than on their actual knowledge of MPX. Regarding knowledge gaps in MPX transmission routes, only 59.0% of participants were aware that MPX is not a sexually transmitted disease. This could be explained by the fact that the majority of surveyed adults rely on social media for information about MPX. However, social media can be flooded with misinformation, particularly in terms of considering MPX as a disease related to homosexuality. Notably, other plausible routes of transmission such as respiratory droplets (31.9%) and vertical transmission (19.2%) were not well-recognized by the respondents. These findings emphasize the importance of raising awareness and knowledge in this area to prevent the spread of the infection.

Regarding the uncovered knowledge gaps, only 11.2% of respondents indicated that the clinical manifestations of HPMX are typically not severe, and only half of them recognized that MPX is not necessarily a deadly disease. Furthermore, only 25% of participants were aware that MPX is not highly transmissible between individuals. Such deficiencies in knowledge regarding the disease could impact the Lebanese population's risk perception, increase their anxiety about potential infection, and lead to misunderstandings about the severity of the disease. Consequently, effective communication regarding the risks of MPX is crucial for public awareness. In addition, only 14.6% of participants were aware that antibiotics should not be used to treat MPX. This issue is concerning given the high levels of antibiotic misuse in Lebanon, where almost half of the population self-medicates with antibiotics, and over 30% of antibiotics are dispensed without a prescription. Another knowledge gap identified pertains to the availability of a specific vaccine, which could impact acceptance of an MPX vaccine once it becomes available. Given that the dearth of knowledge concerning the transmission, treatment, and symptoms can adversely affect disease control, it is recommended to increase the population’s knowledge and awareness to successfully prevent the disease from spreading further.

In terms of socio-demographic factors associated with overall knowledge level, it was found that female gender, increased age, and living in rural areas were negatively associated with a good level of knowledge. A higher level of knowledge about MPX among males was also reported by a recent study conducted in Iraq among the general population. However, our results are inconsistent with those reported in previous research conducted in other countries, such as Jordan, Saudi Arabia, and Indonesia [16, 42, 45]. Although the infection could affect anyone, the majority of MPX cases in this outbreak were recorded among men. Based on this evidence, males may consider themselves more concerned about the disease and have a higher risk perception of possible infection by MPX. This could instigate fears and concerns, and as a result, they will seek more information about the subject, which in turn will improve their knowledge. This study showed that participants aged 50 years and above had higher odds of having a poor knowledge score compared to younger participants. In fact, new media and mass media play an increasingly prominent role in health information. In addition, the internet represents the main source for seeking information about MPX. Hence, the younger population’s familiarity with internet services compared to the older population offers them better access to information about MPX, which in turn raises their awareness of this subject. Of note, older people usually rely on traditional sources to obtain health information. Our results are consistent with the findings of a study conducted in Indonesia.

Regarding residential location, our results showed that it has the potential to affect knowledge level, as participants living in rural areas have a higher likelihood of poor knowledge regarding MPX compared to those residing in urban areas. At present, the internet is the main driver of information, and rural areas are typically less developed, often suffering from limited access and weak internet connections. This can limit the access of people residing in rural areas to information, which could lead to poor knowledge levels. While previous research has reported no significant differences by place of residence, several studies on knowledge of diseases have detected low knowledge levels in rural areas, such as a recent study conducted in Saudi Arabia [16, 63]. Our results highlight the necessity to develop rural publicity and education methods based on the characteristics of rural populations.

However, participants who were married, had higher educational levels, worked in the medical field, had chronic diseases/immunodeficiencies, and had moderate/high economic situations were more likely to have a good knowledge score compared to their counterparts. Highly educated individuals often hold better-paying positions and have access to authoritative information from scientists and other experts. They also frequently participate in and read scientific journals, which allows them to educate themselves better. The higher odds of better knowledge among married participants compared to unmarried ones were expected as married people usually exhibit a higher sense of responsibility and bear the role of protecting their family members and ensuring their well-being. Our results concerning the better level of knowledge among participants with chronic disease or immunodeficiency compared to those without could be explained by the fact that people with chronic diseases are more concerned about their health status. Therefore, they receive preventive treatment of the disease in the early stage and may have regular checkups with their healthcare provider, who could sensitize them regarding MPX.

In terms of occupation, it was presumed that participants working in the medical field were a knowledgeable group, particularly in health-related topics compared with other groups of the population. However, several studies reported that even healthcare providers in non-endemic countries lacked knowledge regarding MPX [16, 42, 45]. Hence, a study exploring the current level of knowledge among Lebanese healthcare workers is recommended.

The study showed that higher education and economic situation play important and positive roles in the knowledge of MPX among Lebanese people. Similar findings in terms of education were found in a recent study conducted in Iraq [64]. Although some studies about knowledge showed that education was unrelated to knowledge [65], our finding was consistent with findings of previous studies on communicable and noncommunicable diseases, which demonstrated that high educational status was correlated with improved disease prevention knowledge [66]. In accordance with our findings, a study assessing knowledge of dengue fever infection among the general population disclosed that high income and high education levels were associated with up to two times better knowledge.

It should be mentioned that highly educated individuals frequently hold better-paying positions which give them better access to reliable information from scientists and other experts [67]. Of note, this difference in knowledge based on the socioeconomic situation emphasized the health inequalities among different socioeconomic groups. This could be explained that participants with poor economic conditions may not pay much attention to health care and may not have the motivation to actively master knowledge [68]. Our results highlighted the need for paying more attention to the public with low education and/or low income and improving their knowledge regarding the disease.

In respect of the MPX source of information, this study highlighted the influence of these sources on the MPX level of knowledge. The majority of participants with a good knowledge level reported scientific journals/research articles on health websites, health authorities, and healthcare providers. Social media and mass media play an increasingly prominent role in health information. However, social media were flooded with misinformation and rumors about MPX. Moreover, health information via the Internet should not be a substitute for healthcare professional experts in emerging infectious diseases. Taken together, the combination of traditional media, new media, and healthcare professional experts would be a better choice to improve the knowledge about emerging and reemerging infectious diseases in the public.

Significant variability in terms of gender, age, geographical areas, socioeconomic situation, and occupation were recorded for the various knowledge domain tackled in this study. Factors such as female gender increased in age and living in rural areas were found negatively associated with a good knowledge level in the majority of domains. On the contrary, working in the medical field, having a good socioeconomic status (moderate/high), and having a higher educational level were associated with a good knowledge level in these domains. Of note, these aforementioned variabilities were also revealed upon comparing the overall level of knowledge level.

### Strengths and limitations

This is the first study to explore the baseline level of information among Lebanese people in the early phase of the emerging MPX outbreak and to disclose knowledge gaps. However, our findings should be interpreted while taking into consideration the timing of the survey. This study can be helpful to tailor well-informed educational and awareness programs aiming to improve the knowledge of MPX emergence among the population based on the disclosed knowledge gaps. Several limitations of this study should be acknowledged in this study such as the cross-sectional design which does not allow us to deduce causality. This study was prone to selection bias due to its being online and with convenience sampling which limits the generalizability of the findings. For example, this study only included people having internet literacy and those who have access to the internet. Face-to-face interviews and longitudinal studies with randomization would be suggested in the future to confirm our results.

### Implications

As more and more cases of MPX are snowballing in different countries, the Lebanese government and population should be prepared for containing a possible outbreak. The findings of this study implied that health authorities should communicate the health information for the MPX disease with a risk of imported infection. Of note, the dissemination of health information prior to the MPX outbreak had a crucial role in preventing the emergence of infectious diseases including MPX. Moreover, the public's sense of social responsibility should be deepened by focusing health information on preparedness, confidence, comprehensive prevention measures, and mitigation techniques. Although media coverage of epidemics frequently serves as the public's primary source of information on MPX, it is insufficient for fostering awareness and beneficial health behaviors and should not be a substitute for trustworthy sources of information such as healthcare professional experts in emerging infectious diseases.

## Conclusion

The current study pointed out a poor knowledge level regarding MPX among the Lebanese population with substantial knowledge gaps in the majority of aspects of MPX knowledge. Our findings stress the urgent need to raise MPX-related awareness and proactively fill the unveiled knowledge gaps. It is suggested that in the promotion of knowledge about the prevention and control of MPX, women, rural residents, families with poor economic conditions, people with no chronic diseases, and lower education should be the key educational objects. In addition, the influence of trustworthy sources of information on improving the knowledge level was revealed. Therefore, using a combination of traditional media, new media, and healthcare professional experts would be a better choice to improve the knowledge about MPX in the public.

## Data Availability

The data sets generated during the current study are not publicly available but are available from the corresponding author on reasonable request. A proposal with a detailed description of study objectives and a statistical analysis plan will be needed for the assessment of requests.
